# Identification of Novel Glial Genes by Single-Cell Transcriptional Profiling of Bergmann Glial Cells from Mouse Cerebellum

**DOI:** 10.1371/journal.pone.0009198

**Published:** 2010-02-12

**Authors:** Samir Koirala, Gabriel Corfas

**Affiliations:** 1 F.M. Kirby Neurobiology Center, Children's Hospital Boston, Boston, Massachusetts, United States of America; 2 Department of Neurology, Harvard Medical School, Boston, Massachusetts, United States of America; 3 Department of Otolaryngology, Harvard Medical School, Boston, Massachusetts, United States of America; University of Washington, United States of America

## Abstract

Bergmann glial cells play critical roles in the structure and function of the cerebellum. During development, their radial processes serve as guides for migrating granule neurons and their terminal endfeet tile to form the glia limitans. As the cerebellum matures, Bergmann glia perform important roles in synaptic transmission and synapse maintenance, while continuing to serve as essential structural elements. Despite growing evidence of the diverse functions of Bergmann glia, the molecular mechanisms that mediate these functions have remained largely unknown. As a step toward identifying the molecular repertoire underlying Bergmann glial function, here we examine global gene expression in individual Bergmann glia from developing (P6) and mature (P30) mouse cerebellum. When we select for developmentally regulated genes, we find that transcription factors and ribosomal genes are particularly enriched at P6 relative to P30; whereas synapse associated molecules are enriched at P30 relative to P6. We also analyze genes expressed at high levels at both ages. In all these categories, we find genes that were not previously known to be expressed in glial cells, and discuss novel functions some of these genes may potentially play in Bergmann glia. We also show that Bergmann glia, even in the adult, express a large set of genes thought to be specific to stem cells, suggesting that Bergmann glia may retain neural precursor potential as has been proposed. Finally, we highlight several genes that in the cerebellum are expressed in Bergmann glia but not astrocytes, and may therefore serve as new, specific markers for Bergmann glia.

## Introduction

The Bergmann glial cell is a type of astroglia that performs a range of important functions in the cerebellum throughout the life of the animal. During development, the radial processes of Bergmann glia provide structural support to the expanding cerebellar plate, and endfeet of these cells adhere together to form a continuous glia limitans overlying the cerebellum [1], [2], [3]. The radial fibers of Bergmann glia also act as essential guide rails for the migration of cerebellar granule cells [4], [5]. Mice with Bergmann glial defects during development have severe abnormalities including pial rupture, disrupted neuronal migration and layering, and altered connectivity [6], [7]. In addition, it has been proposed that Bergmann glia also contribute to the elaboration of Purkinje cell dendrites [8], [9], [10] and the stabilization of synaptic connections onto these neurons [11].

After completion of cerebellar morphogenesis, Bergmann glia remain important for structural support [12], [13], but also serve additional roles in synapse maintenance, function and plasticity [14], [15], [16], [17]. Bergmann glial processes ensheathe synapses on Purkinje cells, and play a supportive role in normal transmission by maintaining a physiological synaptic microenvironment. This involves buffering of ions, uptake of neurotransmitters, and production of glutamine, which neurons then convert to glutamate [16], [18], [19]. Recent studies suggest that Bergmann glia may also play more active roles at synapses. These cells respond to synaptic activity and locomotor behavior with Ca^++^ elevations *in vivo* [20], and may in turn modulate synaptic transmission, synaptic plasticity, and blood perfusion changes around synapses [20], [21], [22].

Despite growing recognition of the diverse roles of Bergmann glia, the molecular mechanisms that mediate these roles remain largely unexplored. As a step toward addressing this gap in knowledge, we analyzed global gene expression in single acutely isolated Bergmann glia from the developing cerebellum (P6), during the peak of granule cell migration along glial fibers, and from the adult (P30), when developmental processes are complete and Bergmann glial processes have ensheathed synapses. We then compared gene expression profiles between Bergmann glia at these two ages, and also with data obtained previously from astrocytes [23] or stem cells [24]. *In situ* hybridization and online gene expression atlases were used to validate Bergmann glial expression of genes of interest. Our analysis shows that although Bergmann glia robustly express many astroglial genes, as expected, there are also genes that appear to be Bergmann glia-specific, and may be useful as new markers and tools to manipulate this cell type. In addition, we identify a number of genes as potential candidates to mediate Bergmann glial roles in maintenance of cerebellar morphology, and in synaptic structure and function. Surprisingly, we also find that Bergmann glia express a large set of genes thought to be expressed specifically in stem cells, suggesting that this glial type may harbor progenitor potential. Together, this information should be useful to future studies of Bergmann glia and glial cells in general.

## Results

### Single Cell Isolation and cDNA Synthesis from Bergmann Glia

**Figure 1:**
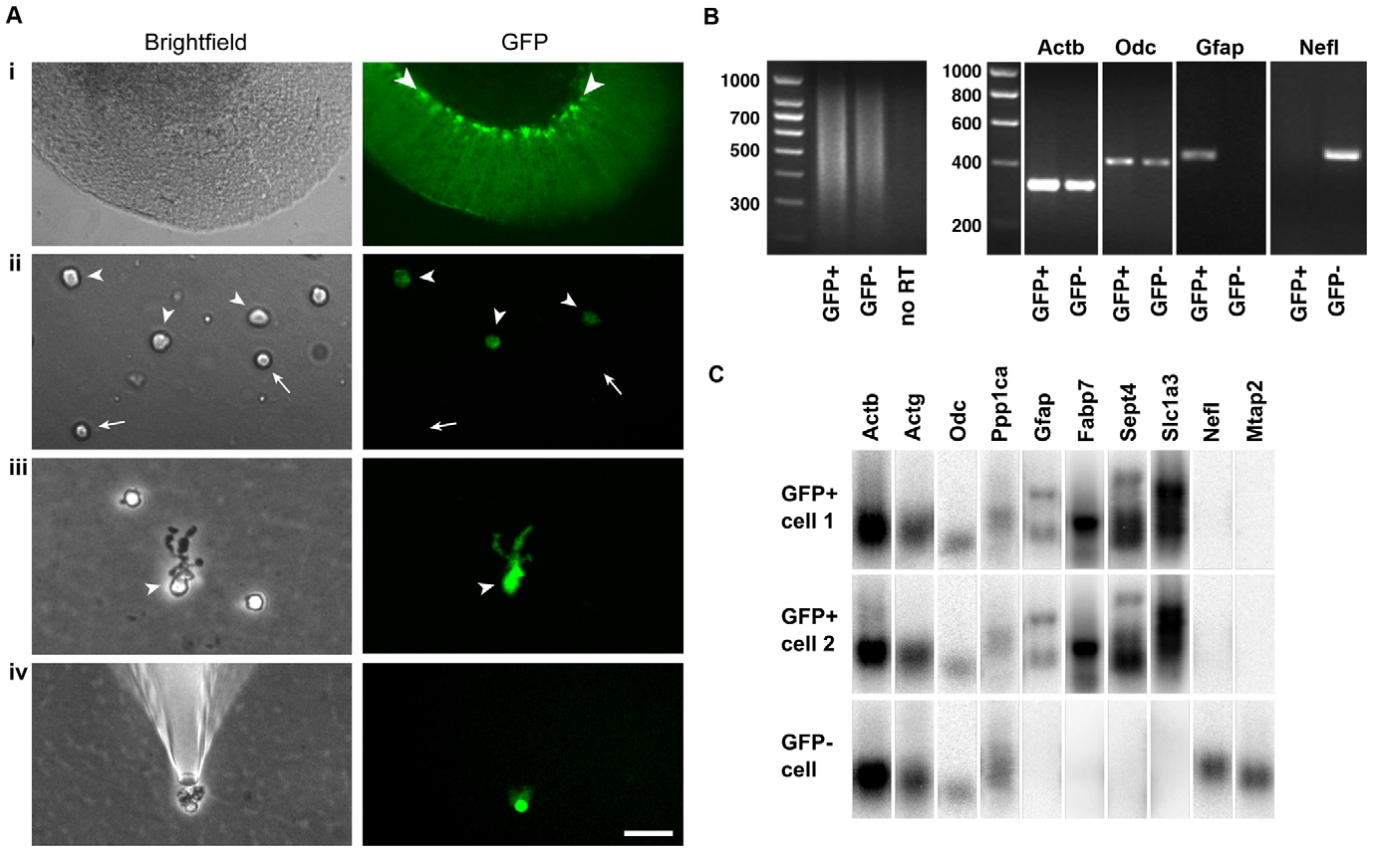
Harvesting of individual Bergmann glia and quality control of single-cell cDNA. **Ai**, a live cerebellar slice obtained from an adult GFAP-GFP transgenic mouse imaged under phase contrast optics (left) and fluorescence illumination (right). The Bergmann glia (see arrowheads) are the cells with the most fluorescence. **Aii**, freshly dissociated cells include putative astrocytes, which are devoid of processes and show relatively weak GFP fluorescence (arrowheads), and putative granule neurons, which have small, round, GFP-negative cell bodies (arrows). **Aiii**, a freshly dissociated Bergmann glia (arrowhead in left panel) can be distinguished from other cells by the bushy processes that emanate from one side of the soma–these are the long Bergmann glial processes that have partially retracted or been sheared off during tissue dissociation. In addition, Bergmann glia display strong GFP fluorescence (arrowhead in right panel), with the mean GFP intensity of their cell bodies 2.9±0.8 fold that of astrocytes; n = 17 cells). **Aiv**, a single Bergmann glia being washed by placement in a new dish containing fresh buffer, before being picked again with a new microelectrode. This step is performed to exclude contaminating cells or mRNAs. Scale bar, 70 µm in **Ai**, 25 µm in **Aii** and **Aiii**, 40 µm in **Aiv**. **B**, left panel, agarose gel electrophoresis of cDNAs generated from single GFP+ and GFP− cells. The gels show that most of the cDNA lies between 300 and 1000 bases. Right panel, agarose gels showing PCR with primer pairs directed towards β-actin (*Actb*), ornithine decarboxylase (*Odc*), *Gfap*, and neurofilament light chain (*Nefl*). The results show that the single cell cDNAs from Bergmann glia (GFP+) and neurons (GFP−) contain both high and low abundance transcripts (*Actb* and *Odc*, respectively). Bergmann glia are positive for the astroglial marker *Gfap* and negative for the neuronal marker *Nefl*, whereas neurons are negative for *Gfap* and positive for *Nefl*. **C**. Southern blot analysis of cDNAs from two putative Bergmann glial cells (GFP+) and a putative neuron (GFP−) shows presence of the high, medium, and two low abundance markers (*Actb*, *Actg*, and *Odc* and *Ppp1ca*, respectively). In addition, the GFP+ cells are positive for *Gfap*, *Fabp7* (BLBP), *Sept4* and *Slc1a3* (GLAST), confirming their glial identity, whereas the GFP− cell lacks all these markers. Conversely, the GFP+ cells are absent for the neuronal markers *Nefl* and *Mtap2* whereas the GFP− cell is positive. These results confirm the preservation of low to high abundance transcripts after the single-cell RT-PCR amplification, and also confirm the cell identity of the Bergmann glia used for microarray analysis.

To examine gene expression in Bergmann glia, we chose a single-cell cDNA library approach [25] using acutely isolated cells from the cerebella of mice expressing GFP under the Glial fibrillary acidic protein (*Gfap*) promoter [26]. As shown Fig. 1Ai, Bergmann glial cell bodies could be easily visualized by their robust GFP expression in a live slice from a GFAP-GFP mouse cerebellum. After gentle dissociation of the tissue, four main types of cells were seen. Most cells were small with round cell bodies and lacked GFP signal (arrows in Fig. 1Aii), and most likely represent granule cells. Another frequent cell type had round cell bodies and relatively weak GFP fluorescence (arrowheads in Fig. 1Aii), almost certainly representing astrocytes. A third cell type, with large cell bodies and no fluorescence, we identified as Purkinje cells (not shown). Finally, a rather infrequent type (<1% of cells) could be readily distinguished from the others by a distinctive “bushy” unipolar morphology and strong GFP expression (arrowheads in Fig. 1Aiii). These putative Bergmann glia and some GFP- cells as controls were harvested individually using glass microelectrodes (Fig. 1Aiv). To minimize the possibility of contamination from other mRNAs, each cell was subjected to a rinse in a new dish with fresh buffer and picked with a new microelectrode before cDNAs were generated using protocols described before [25], [27], [28]. In line with these protocols, single cell RT-PCR amplification generated cDNAs of 300–1000 base pairs (Fig. 1B, left panel). The single cell cDNAs were then subjected to rigorous quality control using PCR (Fig. 1B, right panels). Single cell cDNA libraries were considered to be of good quality if they were positive for high (β-actin or *Actb*) and low abundance (ornithine decarboxylase or *Odc*) markers, confirming that mRNAs of widely varying abundance were preserved during the RT-PCR amplification. High quality cDNAs of GFP+ and GFP− cells were then further characterized. GFP+ cells were positive for *Gfap* but negative for neurofilament light chain (*Nefl*), confirming that they were glia and that the samples were free of contaminating neuronal mRNA. GFP− cells were negative for *Gfap* and positive for *Nefl*, indicating they were most likely neurons. Finally, an additional quality control step was performed using Southern blot analysis (Fig. 1C). GFP+ and GFP− cells were positive for high, medium, and two low abundance transcripts (*Actb*, high; γ-actin or *Actg*, medium; *Odc* and protein phosphatase 1cα or *Ppp1ca*, low), indicating good amplification. GFP+ cells were positive for *Gfap*, fatty acid binding protein 7 or *Fabp7* (also called brain lipid binding protein or BLBP), *Sept4* (Septin 4) and the glutamate transporter, *Slc1a3* (GLAST), confirming that they were astroglial cells. They were negative for the neuronal markers *Nefl* and microtubule-associated protein 2 (*Mtap2*). Conversely, GFP− cells were negative for glial markers but positive for neuronal ones, confirming their identity as neurons.

### Purity and Accuracy of Expression Profiles of Individual Bergmann Glia

**Figure 2:**
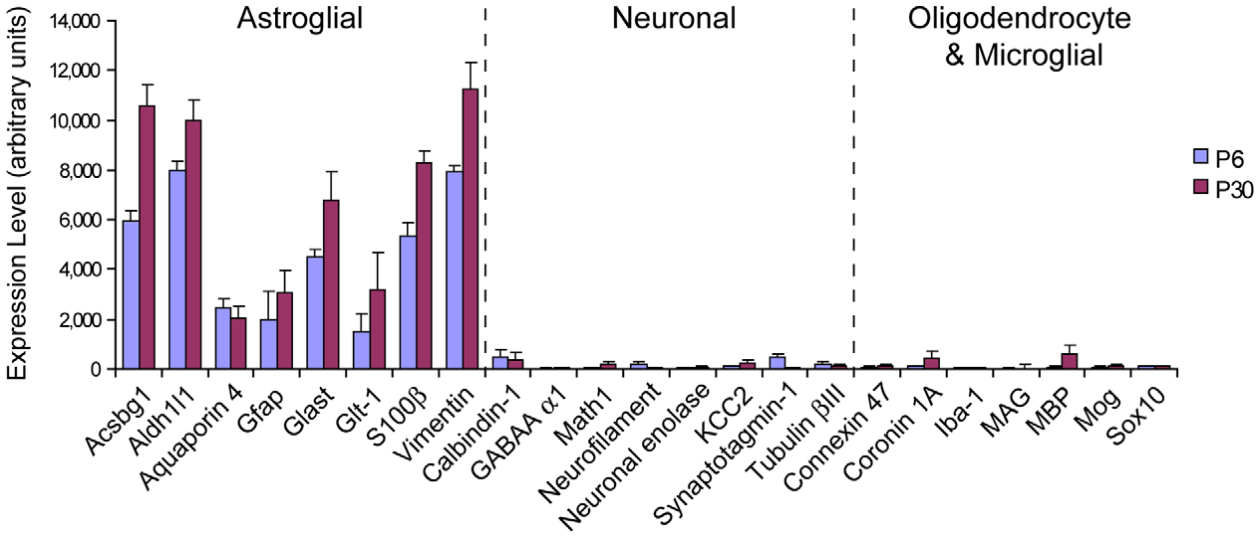
Expression profiles of cell type control genes confirm purity of Bergmann glial cDNA generated by single cell RT-PCR. Mean expression levels of well established markers for astroglia, neurons, oligodendrocytes and microglia were analyzed in the transcriptional profiles of ten Bergmann glia (five each from P6 and P30) using GeneSpring GX 7.3 software. For genes represented by multiple probe sets, the averaged expression of all probe sets were used. Astroglial genes were robustly expressed whereas markers of other cell types were absent or extremely low, confirming the astroglial identity of Bergmann glia and the absence of contaminating mRNAs from other cell types during cell harvesting. Error bars represent ± SEM.

To examine the global transcriptional profiles of Bergmann glia, the amplified cDNA generated from five P6 and five P30 cells were individually hybridized to Affymetrix 430 2.0 Mouse Expression Arrays. On average, 31.7±1.5% of the 45101 probe sets per array showed positive expression at P6, and 27.5±2.3% at P30. Expression profiles of all the Bergmann glia demonstrated high levels of expression of known astrocyte-specific genes [23], consistent with their long-held classification as specialized astroglia (Fig. 2). In contrast, the expression of several genes considered to be markers for neurons, oligodendrocytes or microglia [23] was low or absent (Fig. 2), confirming that the samples were indeed free from contaminating mRNAs from other cell types.

**Figure 3:**
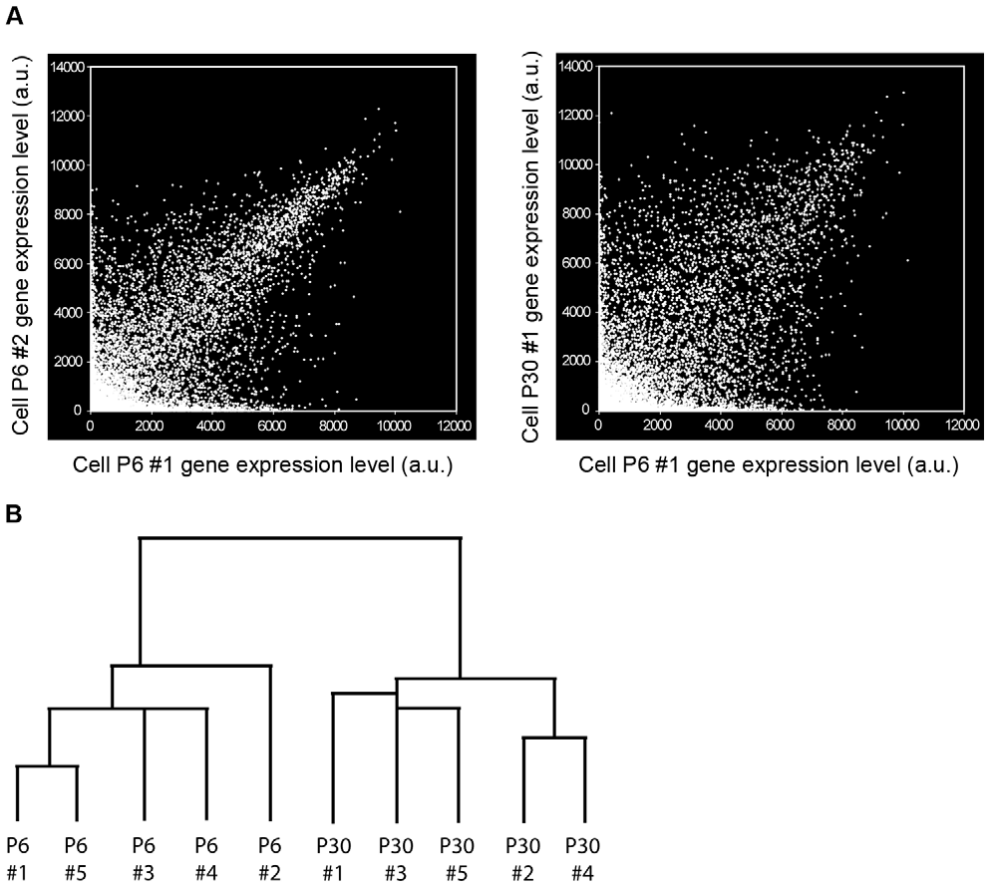
Single cell RT-PCR of individual Bergmann glia is sufficiently accurate for comparison of expression profiles by age. **A**, Scatter plots of raw gene expression level compared between two cells from the same age (P6-1 vs. P6-2; left panel) and between ages (P6-1 vs. P30-1; right panel) (a.u.: arbitrary units). The samples from the same age show high similarity (mean within-age correlation coefficient = 0.81), which is comparable to what is reported between individual cells of a glioblastoma cell line (0.86) [25]. The high level of concordance between samples of the same age increases the reliability of comparisons between ages. Samples of different ages are significantly more divergent (mean P6 vs. P30 correlation coefficient = 0.66). **B**, Dendrogram and sample clustering of individual Bergmann glia. Unsupervised hierarchical clustering based on overall gene expression profiles reveals two distinct clusters corresponding to the two ages, P6 and P30. This suggests that the samples from the two ages do indeed represent two statistically distinct populations suitable for valid comparison.

To determine the level of heterogeneity between samples of the same and different ages, we performed two analyses. Pair-wise comparisons of all individual samples showed that cells from the same age are more similar than between ages (same age: mean correlation coefficient = 0.8 for P6, 0.82 for P30; between ages: mean correlation coefficient = 0.66; representative samples shown in Fig. 3A). The similarity between cells of the same age is comparable to that reported between individual cells of a glioblastoma cell line (mean correlation coefficient = 0.86; [25], using very similar techniques, suggesting that Bergmann glia from a particular age and sagittal location (vermis) are quite homogeneous. The similarity between the two ages (mean correlation coefficient = 0.66) is significantly higher than for disparate cell types (for example, olfactory epithelium neurons vs. heart cells, mean correlation coefficient = 0.42; [25], as expected for cells of the same type. Unsupervised hierarchical clustering of the samples indicated that cells were more distinct between ages than within each age (Fig. 3B). Taken as a whole, these results affirm the cell-type specificity and reproducibility of single cell expression profiling, and the validity of comparisons between ages.

### Identification of Genes that Are Developmentally Regulated in Bergmann Glia

Genes expressed differentially between P6 and P30 were identified using two criteria: those with a greater than 3-fold difference in normalized expression between ages, and those that were flagged “present” at one age and “absent” at the other. In total, 435 genes were found to fit the criteria for P6>P30 expression (Table S1), and 137 fit the criteria for P30>P6 expression (Table S2). Of these, the top twenty most differentially expressed probe sets determined using the two approaches are shown in Tables 1 and 2 for P6>P30 and in Tables 3 and 4 for P30>P6. Genes in all tables were categorized and annotated based on functional information obtained in online databases and/or previous studies.

**Table 1:** Top 20 probe-sets showing largest fold-difference in expression (P6>P30).

Probe ID	Gene Symbol	Fold Change	Description	Functional Classification
1448182_a_at	Cd24a	317.5	CD24a antigen	cell cycle regulation
1456010_x_at	Hes5	264.3	hairy and enhancer of split 5 (Drosophila)	regulation of transcription
1416200_at	Il33	248.6	interleukin 33	cytokine signaling
1435612_at	Opcml	233	opioid binding protein/cell adhesion molecule-like	cell adhesion or repulsion
1454778_x_at	Rps28	200.7	Ribosomal protein S28, mRNA	component of ribosome
1421100_a_at	Dab1	181.8	disabled homolog 1 (Drosophila)	regulation of cell adhesion
1455085_at	1700086L19Rik	177.9	RIKEN cDNA 1700086L19 gene	function unknown
1448842_at	Cdo1	170.9	cysteine dioxygenase 1, cytosolic	metabolic functions
1415945_at	Mcm5	164.7	minichromosome maintenance deficient 5	cell cycle regulation
1436808_x_at	Mcm5	156.2	minichromosome maintenance deficient 5	cell cycle regulation
1419123_a_at	Pdgfc	143.6	platelet-derived growth factor C	growth factor signaling
1429372_at	Sox11	140.7	SRY-box containing gene 11	regulation of transcription
1418310_a_at	Rlbp1	140	retinaldehyde binding protein 1	transporter activity
1436505_at	Ppig	137	peptidyl-prolyl isomerase G (cyclophilin G)	protein folding
1460011_at	Cyp26b1	135.1	cytochrome P450, family 26, b1	morphogenesis/patterning
1426307_at	Cyb5r4	129.2	cytochrome b5 reductase 4	metabolic functions
1424010_at	Mfap4	121.3	microfibrillar-associated protein 4	cell adhesion or repulsion
1423763_x_at	Rps28	117.7	Ribosomal protein S28, mRNA	component of ribosome
1428466_at	Chd3	114.9	chromodomain helicase DNA binding protein 3	chromatin remodeling
1425052_at	Isoc1	107.8	isochorismatase domain containing 1	metabolic functions

**Table 2:** Top 20 probe-sets present at P6 and absent at P30 (ranked by P values).

Probe ID	Gene Symbol	P value	Description	Functional classification
1448842_at	Cdo1	5.28E-06	cysteine dioxygenase 1, cytosolic	metabolic functions
1454778_x_at	Rps28	2.89E-05	Ribosomal protein S28, mRNA	component of ribosome
1448182_a_at	Cd24a	6.02E-05	CD24a antigen	cell cycle regulation
1433928_a_at	Rpl13a	6.02E-05	ribosomal protein L13a	component of ribosome
1435612_at	Opcml	0.0001	opioid binding protein/cell adhesion molecule-like	cell adhesion or repulsion
1460011_at	Cyp26b1	0.00012	cytochrome P450, family 26, b1	morphogenesis/patterning
1418310_a_at	Rlbp1	0.00012	retinaldehyde binding protein 1	transporter activity
1455085_at	1700086L19Rik	0.00012	RIKEN cDNA 1700086L19 gene	function unknown
1433935_at	AU020206	0.00016	expressed sequence AU020206	function unknown
1419123_a_at	Pdgfc	0.00016	platelet-derived growth factor C	growth factor signaling
1449013_at	Eef2k	0.00016	eukaryotic elongation factor-2 kinase	protein biosynthesis
1452499_a_at	Kif2a	0.00021	kinesin family member 2A	microtubule based motor
1454867_at	Mn1	0.00025	meningioma 1	cell growth and proliferation
1416200_at	Il33	0.00035	interleukin 33	cytokine signaling
1420329_at	4930455C21Rik	0.00036	RIKEN cDNA 4930455C21 gene	transporter activity
1416107_at	Nsg2	0.00036	neuron specific gene family member 2	intracellular signaling
1417038_at	Sept9	0.00045	septin 9	cell cycle regulation
1421100_a_at	Dab1	0.00053	disabled homolog 1	regulation of cell adhesion
1456010_x_at	Hes5	0.00063	hairy and enhancer of split 5	regulation of transcription
1424275_s_at	Trim41	0.00085	tripartite motif-containing 41	function unknown

**Table 3:** Top 20 probe-sets showing largest fold-difference in expression (P30>P6).

Probe ID	Gene Symbol	Fold change	Description	Functional classification
1438044_at	1700047M11Rik	293.1	RIKEN cDNA 1700047M11 gene	function unknown
1434264_at	Ank2	266.8	ankyrin 2, brain	synapse structure and function
1418288_at	Lpin1	169.8	lipin 1	metabolic functions
1439568_at	Greb1	159.4	gene regulated by estrogen in breast cancer protein	electron transport
1430268_at	9630005C17Rik	154.6	RIKEN cDNA 9630005C17 gene	function unknown
1448428_at	Nbl1	143.3	neuroblastoma, suppression of tumorigenicity 1	cell cycle regulation
1427284_a_at	Ttpa	137.4	tocopherol (alpha) transfer protein	transporter activity
1434121_at	Lgi4	126.1	leucine-rich repeat LGI family, 4	cell-cell interaction
1434265_s_at	Ank2	108.7	ankyrin 2, brain	synapse structure and function
1435436_at	Epas1	101.2	endothelial PAS domain protein 1	regulation of transcription
1436470_at	Rims2	97.07	regulating synaptic membrane exocytosis 2	exocytosis
1456642_x_at	S100a10	95.89	S100 calcium binding protein A10 (calpactin)	intracellular signaling
1421841_at	Fgfr3	95.79	fibroblast growth factor receptor 3	growth factor signaling
1456523_at	C77713	95.74	expressed sequence C77713	function unknown
1416762_at	S100a10	85.57	S100 calcium binding protein A10 (calpactin)	intracellular signaling
1451718_at	Plp1	80.05	proteolipid protein (myelin) 1	myelin-associated
1438193_at	Nrxn3	72.57	neurexin III	synapse structure and function
1433788_at	Nrxn3	69.35	neurexin III	synapse structure and function
1423602_at	Traf1	67.72	Tnf receptor-associated factor 1	intracellular signaling
1417220_at	Fah	65.95	fumarylacetoacetate hydrolase	hydrolase activity

**Table 4:** Top 20 probe-sets present at P30 and absent at P6 (ranked by P values).

Probe ID	Gene Symbol	P value	Description	Functional classification
1434265_s_at	Ank2	3.60E-06	ankyrin 2, brain	synapse structure and function
1434264_at	Ank2	3.60E-06	ankyrin 2, brain	synapse structure and function
1438044_at	1700047M11Rik	7.65E-05	RIKEN cDNA 1700047M11 gene	function unknown
1436173_at	Dlc1	0.00087	Deleted in liver cancer 1 (Dlc-1)	intracellular signaling
1451784_x_at	H2-D1	0.00087	histocompatibility 2, D region locus 1	MHC class I receptor
1439568_at	Greb1	0.00087	gene regulated by estrogen in breast cancer protein	electron transport
1423523_at	Aass	0.0011	aminoadipate-semialdehyde synthase	metabolic functions
1438193_at	Nrxn3	0.0011	neurexin III	synapse structure and function
1420709_s_at	Dao1	0.0011	D-amino acid oxidase 1	D-amino acid pathway
1425545_x_at	H2-D1	0.00151	histocompatibility 2, D region locus 1	MHC class I receptor
1436205_at	Nfasc	0.00186	neurofascin	synapse structure and function
1417220_at	Fah	0.00186	fumarylacetoacetate hydrolase	hydrolase activity
1425567_a_at	Anxa5	0.00189	annexin A5	intracellular signaling
1434121_at	Lgi4	0.00191	leucine-rich repeat LGI family, 4	intracellular signaling
1417629_at	Prodh	0.00194	proline dehydrogenase	metabolic functions
1420545_a_at	Chn1	0.00346	chimerin (chimaerin) 1	intracellular signaling
1427284_a_at	Ttpa	0.00346	tocopherol (alpha) transfer protein	ion transport
1436470_at	Rims2	0.00346	regulating synaptic membrane exocytosis 2	exocytosis
1418288_at	Lpin1	0.00346	lipin 1	metabolic functions
1421841_at	Fgfr3	0.00346	fibroblast growth factor receptor 3	growth factor signaling

We found that the set of genes expressed more highly at P6 is enriched in molecules known or predicted to be involved in cell growth and/or proliferation, cell cycle regulation, protein biosynthesis and other metabolic pathways, RNA processing and transport, and transcriptional regulation (including a large number of transcription factors) (Table S1). This suggests that at P6 Bergmann glia are in a state of active metabolism and growth, and some of them may potentially still be undergoing proliferation. The higher expression levels of ribosomal genes is also indicative of cells in a state of growth [29], [30], consistent with the postnatal extension and elaboration of Bergmann glial processes.

On the other hand, the genes enriched at P30 include a different set of functional categories, namely molecules known or predicted to be involved in maintaining synapse structure and function, regulating exocytosis, forming gap junctions, and mediating molecular transport (including ion, protein, and carbohydrate transporters) (Table S2). This result suggests that a key role of Bergmann glia in the adult cerebellum is to support/modulate synaptic function, and identifies some potential molecular players that may mediate this role. Interestingly, many of the differentially expressed genes in both sets (P6>P30 and P30>P6) have not previously been reported to be present in astroglial cells (Table 5), based on a search of the literature and exclusion of astrocyte-enriched genes listed by Cahoy and colleagues [23]. Further study of these genes, some of which are discussed below, may provide new insight into molecular mechanisms underlying Bergmann glial functions.

**Table 5:** Differentially expressed (P6 vs. P30) genes in Bergmann glia not previously known to be present in astroglia.

Gene Symbol	Description	Functional Classification	Age
Hs6st1	heparan sulfate 6-O-sulfotransferase 1	cell adhesion or guidance	P6
Sema6a	semaphorin 6A	cell adhesion or guidance	P6
Cd24a	CD24a antigen	cell cycle regulation	P6
Atxn2	ataxin 2	cell growth and/or proliferation	P6
Asxl1	additional sex combs like 1 (Drosophila)	chromatin remodeling	P6
Nnat	neuronatin	function unknown	P6
Neurl2	neuralized-like 2	intracellular signaling (notch)	P6
Shroom3	shroom family member 3	morphogenesis/pattern formation	P6
Shfm1	split hand/foot malformation (ectrodactyly) type 1	protein processing	P6
Adamts12	a disintegrin-like metallopeptidase, thrombospondin 1 motif, 12	proteolysis and/or cell-ECM interaction	P6
Solh	small optic lobes homolog	proteolysis and/or cell-ECM interaction	P6
Bmi1	Bmi1 polycomb ring finger oncogene	regulation of transcription	P6
Myef2	myelin basic protein expression factor 2, repressor	regulation of transcription	P6
Zfp131	zinc finger protein 131	regulation of transcription	P6
Zfp260	zinc finger protein 260	regulation of transcription	P6
Zfp414	zinc finger protein 414	regulation of transcription	P6
Zfp532	zinc finger protein 532	regulation of transcription	P6
Zfp560	zinc finger protein 560	regulation of transcription	P6
Zfp651	zinc finger protein 651	regulation of transcription	P6
Zfp704	zinc finger protein 704	regulation of transcription	P6
Cyp26b1	cytochrome P450, family 26, subfamily b, polypeptide 1	retinoic acid signaling	P6
Smn1	survival motor neuron 1	RNA synthesis or processing	P6
Snapap	SNAP-associated protein	vesicle exocytosis	P6
Syt16	synaptotagmin XVI	vesicle exocytosis	P6
Hap1	huntingtin-associated protein 1	vesicle transport	P6
Dlc1	Deleted in liver cancer 1 (Dlc-1)	cell adhesion or repulsion	P30
Gpr89	G protein-coupled receptor 89	GPCR signaling	P30
Chn1	chimerin (chimaerin) 1	intracellular signaling	P30
Plp1	proteolipid protein (myelin) 1	myelin-associated	P30
Nfasc	neurofascin	synapse structure and function	P30
Nrxn3	neurexin III	synapse structure and function	P30
Rims2	regulating synaptic membrane exocytosis 2	transporter activity/exocytosis	P30

In addition to individual genes, we also sought to identify signaling and metabolic pathways that are preferentially active in Bergmann glia at P6 or at P30. To do this, we utilized the Ingenuity Pathway Analysis (IPA) tool from Ingenuity Systems, a resource based on a curated list of all canonical signaling and metabolic pathways [31]. IPA analysis of the differentially expressed gene sets in our data identified nine signaling or metabolic pathways statistically enriched at P6 and nine enriched at P30 (Table 6). The pathways enriched at P6 include the Notch, TGF-β and Wnt/β-catenin signaling pathways, which have key developmental roles in cell fate determination, cell growth, proliferation and maturation. The Notch pathway has been shown to be important for Bergmann glial specification and maturation [32], [33], [34], but the potential roles of the other pathways we have identified remain untested. At P30, the statistically enriched signaling pathways included the glutamate receptor signaling pathway, underscoring a role for Bergmann glia in synapse function/modulation. Unexpectedly, another enriched pathway is the embryonic stem cell pluripotency pathway, a finding that we discuss in greater detail below.

**Table 6:** Signaling and metabolic pathways enriched in Bergmann glia.

Enriched at P6	
Pathway	P-value
Aryl Hydrocarbon Receptor Signaling	2.0E-03
Notch Signaling	5.4E-03
Pantothenate and CoA Biosynthesis	5.5E-03
Cell Cycle: G2/M DNA Damage Checkpoint Regulation	7.9E-03
Glycosphingolipid Biosynthesis - Ganglioseries	1.5E-02
CD27 Signaling in Lymphocytes	2.0E-02
Mitotic Roles of Polo-Like Kinase	2.4E-02
TGF-beta Signaling	2.4E-02
Wnt/beta-catenin Signaling	3.6E-02

Enriched at P30	
Pathway	P-value
Glycerophospholipid Metabolism	1.5E-03
Sphingosine-1-phosphate Signaling	6.0E-03
Glutamate Receptor Signaling	8.3E-03
Human Embryonic Stem Cell Pluripotency	9.5E-03
Inositol Metabolism	1.0E-02
Phospholipid Degradation	1.8E-02
Glycerolipid Metabolism	2.5E-02
Oncostatin M Signaling	2.9E-02
Thrombin Signaling	4.3E-02

We were also surprised to find that at P6 Bergmann glia express a number of genes that have been traditionally thought of as neuron-specific (see Table S3). While we cannot completely exclude the possibility of contamination by some neuronal mRNAs, our quality control analysis (Fig. 2) argues against this, as does the finding that some of these are also enriched in cortical astrocytes [23]. We therefore believe that these genes are expressed by Bergmann glia in addition to neurons, but that the level of expression in glia may be significantly lower than in neurons, causing them not to have been detected in the glia by other techniques such as *in situ* hybridization. We believe one of the strengths of our analysis is that we have not subtracted out genes thought to be specific to other cell types from our microarray data, and therefore we can detect genes that may be present in multiple cell types, even if they are more abundant in other cells than in Bergmann glia.

### Analysis of Genes Abundantly Expressed in Postnatal Bergmann Glia (P6 and P30) and Identification of Novel Cell-Specific Markers

To gain further insight into the molecular makeup of Bergmann glia in the postnatal cerebellum, we also searched for genes that are expressed at both P6 and P30 at moderate to high levels (raw signal values >2000) (Table S4). As expected, this extensive list includes many astroglial markers such as vimentin, S100β, aquaporin 4 and aldehyde dehydrogenase 1 family, member L1 (*Aldh1L1*) [23]. It also includes genes not previously known to be expressed in astroglia, some of which are listed in Table 7.

**Table 7:** Genes with abundant Bergmann glial expression (at both P6 and P30) that were not previously known to be present in astroglia.

Gene Symbol	Description	Functional Classification	Age
Cdh22	cadherin 22	cell adhesion or ECM binding	P6, P30
Celsr2	cadherin EGF LAG seven-pass G-type receptor 2	cell adhesion or ECM binding	P6, P30
Cbx3	chromobox homolog 3	chromatin remodeling	P6, P30
Gpr153	G protein-coupled receptor 153	GPCR signaling	P6, P30
Gpsm1	G-protein signalling modulator 1 (AGS3-like, C. elegans)	GPCR signaling	P6, P30
Kcnb1	potassium voltage gated channel, Shab-related subfamily	ion channel or receptor activity	P6, P30
Edf1	endothelial differentiation-related factor 1	regulation of transcription	P6, P30
Tcfl5	transcription factor-like 5 (basic helix-loop-helix)	regulation of transcription	P6, P30
Stx5a	syntaxin 5A	SNARE receptor activity	P6, P30
Stx8	syntaxin 8	SNARE receptor activity	P6, P30
Kcnb1	potassium voltage gated channel, Shab-related subfamily	ion channel or receptor activity	P6, P30
Cbx3	chromobox homolog 3	chromatin remodeling	P6, P30

While examining the set of abundantly expressed genes, we found, surprisingly, that Bergmann glia express mRNA for the myelin protein peripheral myelin protein (Pmp22) and for myelin protein zero-like 1 (*Mpzl1*), which may be important for myelination [35] (Table S4). Furthermore, in the adult, Bergmann glia also express mRNAs for proteolipid protein 1 (*Plp1*), the predominant component of CNS myelin, and leucine-rich repeat LGI family, member 4 (*Lgi4*), which is important for myelination in the PNS [36] (Table S2). These findings were supported by *in situ* hybridization data from the Allen Brain Atlas (Fig. S1): although the strongest expression of *Pmp22* and *Plp1* in the adult is in putative oligodendrocytes (arrows in Fig. S1, top and second row), there is less intense but still significant staining in the Purkinje cell layer (PCL) consistent with expression in Bergmann glia (arrowheads). *Lgi4* (Fig. S1, third row) and *Mpzl1* (arrowheads in Fig. S1, bottom row) also show labeling consistent with expression in Bergmann glia. Since Bergmann glia play no known role in myelination, it is unknown what alternate function these genes may serve in these cells. Our data also indicated that Bergmann glia express agrin *in vivo* at P6 and P30 (Table S4), similar to what been shown in astrocytes *in vitro* [37]. Whether agrin synthesized by Bergmann glia plays any role in synapse formation or maintenance *in vivo* merits further investigation.

Analysis of *in situ* hybridization data for genes we found expressed in adult Bergmann glia identified several candidates that we believe may serve as selective markers for Bergmann glia. Within the adult cerebellum, the expression of four genes, leucine zipper protein 2 (*Luzp2*), G protein-coupled receptor 89 (*Gpr89*), leucine-rich repeat LGI family, member 4 (*Lgi4*), and Growth and differentiation factor 10 (*Gdf10*) appears to be restricted solely to the Purkinje cell layer (Fig. S2); and the cellular expression patterns of these genes within the PCL closely match those of well established astroglial markers expressed by Bergmann glia (Fig. S3). However, unlike most currently used markers that also label cerebellar astrocytes, these four genes appear to be completely specific to Bergmann glia. *Gdf10* is particularly noteworthy because its specific expression in the Purkinje cell layer had been reported before [38], but was thought, we believe in error, to be in Lugaro cells rather than in Bergmann glia.

### Gpr126, an Adhesion GPCR Expressed Specifically in Developing Bergmann Glia

**Figure 4:**
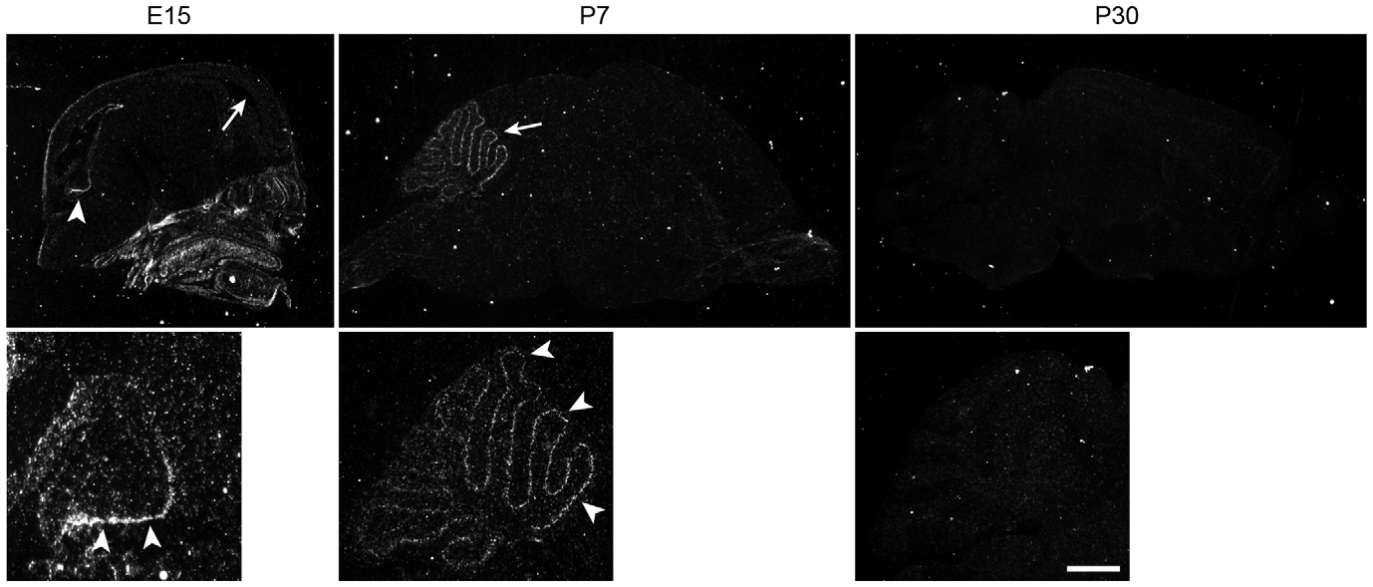
Identification of a developmentally regulated GPCR that is Bergmann glia-specific in the cerebellum. *in situ* hybridization with a ^33^P-labeled probe for *Gpr126*, a little known GPCR of the adhesion family, reveals signal specifically in the Purkinje cell layer at P7 (arrows, middle panels), consistent with expression in Bergmann glia. *Gpr126* expression is developmentally regulated, and becomes undetectable in the adult (right panel). Unlike most classic Bergmann glial markers, which are also expressed by cortical radial glia, *Gpr126* is specific to Bergmann glia and not detected in cortical radial glia at E15 (arrow in top left panel). Labeling is seen in the ventricular zone of the developing cerebellar anlage at E15 (arrowheads in left panels), suggesting that *Gpr126* may be expressed in progenitors of Bergmann glia. All sections are oriented with rostral to the right. Scale bar, upper panels: left, 3 mm; center, 2 mm; right, 2.5 mm; lower panels: left, 100 µm; center, 1 mm; right, 1.4 mm.

GPCRs were one of the gene families of particular interest to us in light of our recent finding that *Gpr56*, an adhesion GPCR, is essential for cortical and cerebellar development [39], [40]. Moreover, few GPCRs have been studied in the context of glial function. Among the many GPCRs we found expressed in Bergmann glia, we were especially intrigued by the very specific spatiotemporal pattern of expression of *Gpr126*, another orphan receptor that is a close relative of *Gpr56*. At P6, *Gpr126* was expressed specifically in the Purkinje cell layer (Fig. 4, center panels). Since RT-PCR analysis of single-cell cDNAs from Purkinje cells showed that *Gpr126* was absent in these neurons (data not shown), the pattern of *GPR126* mRNA is consistent with expression by Bergmann glial cells. As predicted by the microarray data, *in situ* hybridization signal for *Gpr126* was no longer detectable in the adult cerebellum (Fig. 4, right panels). At E15, *Gpr126* was present in the cerebellar ventricular zone (arrowheads in Fig. 4, left panels), where precursors of cerebellar neurons and glia–including Bergmann glia–are located. In contrast, *Gpr126* was absent in the forebrain ventricular zone at E15 (Fig. 4, arrow in left panel), indicating that it does not play a role in cortical radial glia at this age. Based on a recent study in zebrafish, regulation of cyclic AMP by *Gpr126* signaling plays a critical role in the initiation of myelination by Schwann cells [41]. Whether *Gpr126* in Bergmann glia also regulates second messenger pathways involved in cell differentiation, or instead regulates cell adhesion similar to other adhesion GPCRs such as *Gpr56*, *Celsr2* and *Celsr3* [39], [42], [43] remains to be examined.

### Bergmann Glia Express Genes Typical of Neural Stem Cells

A striking finding of our microarray analysis is that Bergmann glia, even in the adult, express numerous genes thought to be expressed specifically by stem cells (Table 8), and show enrichment of the embryonic stem cell pluripotency pathway (Table 6). Of the 220 genes that [24] identified as a core set of “stemness” genes common to multiple types of stem cells but not found in differentiated cells, 26.8% (60/220) are expressed by Bergmann glia at P6 (Table 8). Remarkably, 18.3% (41/220) remain expressed at P30. In addition, 26.3% (647/2458) of neural stem cell genes identified in the same study are also present in Bergmann glia at one or both ages (data not shown). These include transcription factors such as *Sox1*, *Sox2*, *Sox9*, *Hes1* and *Hes5*, which play important roles in cell proliferation and in maintaining neural stem cell identity. These findings support the intriguing hypothesis [44] that Bergmann glia may be a source of the newly described neural stem cells in the adult cerebellum [45], [46]. Our observation in Bergmann glia is also consistent with reports of progenitor cell-like gene expression in Muller Glia of the retina [44], [47].

**Table 8:** Stem cell-enriched genes expressed in Bergmann glia.

Gene Symbol	Description	Functional Classification
Pls3	plastin 3 (T-isoform)	actin binding
Aldh7a1	aldehyde dehydrogenase family 7, member A1	aldehyde metabolism
Pdcd2	programmed cell death 2	apoptosis
Cbr3	carbonyl reductase 3	Arachidonic acid metabolism
Rcn1	reticulocalbin 1	calcium ion binding
Tbrg1	transforming growth factor beta regulated gene 1	cell cycle regulation
Fhl1	four and a half LIM domains 1	cell growth and differentiation
Msh2	mutS homolog 2	DNA repair
Acadm	acyl-Coenzyme A dehydrogenase, medium chain	electron transport
Trip6	thyroid hormone receptor interactor 6	electron transport
Txndc9	thioredoxin domain containing 9	electron transport
Txnl1	thioredoxin-like 1	electron transport
2410015N17Rik	RIKEN cDNA 2410015N17 gene	function unknown
2410022L05Rik	RIKEN cDNA 2410022L05 gene	function unknown
AW549877	expressed sequence AW549877	function unknown
Jagn1	jagunal homolog 1	function unknown
Sh3d19	SH3 domain protein D19	function unknown
Gsta4	glutathione S-transferase, alpha 4	Glutathione metabolism
Pigx	phosphatidylinositol glycan anchor biosynthesis, class X	GPI-anchor biosynthesis
Tbc1d15	TBC1 domain family, member 15	GTPase activator activity
Pla2g6	phospholipase A2, group VI	lipid catabolism
Sfrs3	splicing factor, arginine/serine-rich 3 (SRp20)	nuclear mRNA splicing
Sfrs6	splicing factor, arginine/serine-rich 6	nuclear mRNA splicing
Hrsp12	heat-responsive protein 12	nuclease activity
Zc3h14	zinc finger CCCH type containing 14, variant 1, mRNA.	nucleic acid binding
Ppa1	pyrophosphatase (inorganic) 1	phosphate metabolism
Nup35	nucleoporin 35	porin activity
Psmd12	proteasome 26S subunit, non-ATPase, 12	proteasome pathway
Tjp1	tight junction protein 1	protein binding
Mrpl17	mitochondrial ribosomal protein L17	protein biosynthesis
Eif2b4	eukaryotic translation initiation factor 2B, subunit 4 delta	protein biosynthesis
Eif3s1	eukaryotic translation initiation factor 3, subunit 1 alpha	protein biosynthesis
Eprs	glutamyl-prolyl-tRNA synthetase	protein biosynthesis
Iars	isoleucine-tRNA synthetase	protein biosynthesis
Mrpl3	mitochondrial ribosomal protein L3	protein biosynthesis
Mrpl34	mitochondrial ribosomal protein L34	protein biosynthesis
Fkbp11	FK506 binding protein 11	protein folding
Fkbp9	FK506 binding protein 9	protein folding
Ppic	peptidylprolyl isomerase C	protein folding
Mpdu1	mannose-P-dolichol utilization defect 1	protein metabolism
Esf1	ESF1, nucleolar pre-rRNA processing protein, homolog	protein processing
Ywhab	tyrosine 3-monooxygenase activation protein, beta	protein targeting
Laptm4a	lysosomal-associated protein transmembrane 4A	protein transport
Pkd2	polycystic kidney disease 2	protein transport
Xpot	exportin, tRNA (nuclear export receptor for tRNAs)	protein transport
Zmat3	zinc finger matrin type 3	regulation of cell growth
Smarcad1	SWI/SNF-related, matrix-associated chromatin regulator 1a	regulation of DNA recombination
Epl2	elongation protein 2 homolog	regulation of JAK-STAT cascade
Cops4	constitutive photomorphogenic homolog, subunit 4	regulation of signaling
Rnf4	ring finger protein 4	regulation of transcription
Tead2	TEA domain family member 2	regulation of transcription
Zfx	zinc finger protein X-linked	regulation of transcription
Gnl2	guanine nucleotide binding protein-like 2 (nucleolar)	ribosome biogenesis
Nol5a	nucleolar protein 5A	ribosome biogenesis
Mphosph10	M-phase phosphoprotein 10	rRNA processing
Gnb1	guanine nucleotide binding protein, beta 1	signal transduction
Crtap	cartilage associated protein	sugar transport
3732413I11Rik	RIKEN cDNA 3732413I11 gene	ubiquitin cycle
Fbxo38	F-box protein 38	ubiquitin cycle
Wbp5	WW domain binding protein 5	WW domain binding

## Discussion

Despite growing evidence of the indispensable roles of glial cells in many aspects of nervous system development and function, much remains unknown about the molecules that mediate these roles, particularly *in vivo*. There is also a lack of specific markers for various subtypes of glial cells, and of tools to manipulate gene expression only in specific subtypes. This has hindered our understanding of the diverse roles of glial cells. Our results from single cell transcriptome analysis of cerebellar Bergmann glia identify numerous novel genes whose role in Bergmann glia, or glia in general, can now be tested in functional contexts. We also identify several genes that appear to be entirely Bergmann glia-specific in the cerebellum. Not only does this confirm that the GFP+ cells we harvested for this study were indeed Bergmann glia and did not inadvertently include astrocytes, but also offers new tools for understanding these important glial cells. In this study we focused mainly on genes not previously characterized in Bergmann glia or glial cells in general. For analysis of all genes that we found expressed in Bergmann glia, our complete data set can be viewed in Table S5, and the original Affymetrix. CEL files can be accessed at the Gene Expression Omnibus (GEO) repository (accession number GSE18617).

Genes identified here as being expressed in Bergmann glia fall into many classes, highlighting the diverse roles of this cell type throughout life. Some of the most salient with respect to cerebellar structure and function include retinoic acid signaling components–e.g. *Cyp26b1*, a regulator of retinoic acid activity - which may play a role in cerebellar patterning [48]; chemoattractive and chemorepulsive molecules (notably semaphorin 4B), which may regulate neuronal migration as well as dendritic outgrowth and synapse development [49], [50], [51]; growth factors and growth factor-like molecules such as Gdf10 [38], [52] and meteorin [53], [54]; molecules for cell-cell communication, particularly gap junction proteins, which have been shown to play critical roles in radial glial proliferation [55] and neuronal migration along radial glial fibers in the cerebral cortex [56]; synapse-associated adhesion molecules such as neurexins, which are important for synapse formation and maintenance [57]; components of the D-serine pathway involved in modulation of NMDA receptor function [58], [59]; and molecules that mediate synaptic function and plasticity, such as glutamate receptors, transporters, and transmembrane AMPAR regulatory proteins (TARPs) [60]. The expression of the enzymes glutamine synthetase and pyruvate carboxylase along with glial glutamate transporters *Slc1a3* (GLAST) and *Slc1a2* (GLT-1) provide further evidence for the involvement of Bergmann glia in the glutamate-glutamine cycle that supports synaptic activity [19], [61]. Similarly, the expression of the lactate synthetic enzymes lactate dehydrogenase A and B (*Ldha* and *Ldhb*), and monocarboxylate transporter 1 (*Mct1*), the main lactate transporter responsible for rapid release of glial lactate, is consistent with the hypothesized glia-neuron lactate shuttle [62].

Our finding that neurexin III is expressed in Bergmann glia is particularly interesting in light of the proposed role of neurexins in the development and maintenance of functional synapses. Neurexins present on the presynaptic membrane are thought to bind neuroligins on the postsynaptic membrane, thereby forming a trans-synaptic link that helps maintain the close apposition of pre- and post-synaptic elements [63]. Originally thought to be presynaptic [64], immuno-electron microscopy has now shown that neurexins are also present postsynaptically [65]. What has been missing in this analysis is the consideration of glial processes, which are also integral components of most CNS synapses and maintained in close proximity to pre-and post-synaptic elements [9], [66]. The expression of neurexin III in Bergmann glia raises the question of whether this molecule plays a role in anchoring glial processes to pre- and post-synaptic elements. Cell type-specific deletion of this gene in astroglial cells can be performed to address this hypothesis.

The role of glia in synaptic function has been reinforced by the finding that astrocytes in cell culture or brain slices can release glutamate [67], [68], which in turn can modulate synaptic transmission and plasticity [69], [70], [71]. However, the actual mechanism of glial glutamate release has remained controversial [72]. There is evidence that astrocytes *in vitro* express components of regulated vesicle exocytosis previously thought to be found only in neurons, including v-glut1/2, SNAP23, Munc18a, and synaptotagmin IV [73], suggesting that astrocytes release glutamate by vesicle exocytosis similar to neurons. However, a recent study [23] reported that acutely isolated mouse astrocytes do not express v-glut1/2, synaptotagmins or synapsin I, and therefore are unlikely to exhibit regulated vesicular glutamate release *in vivo*. Our finding that acutely isolated adult Bergmann glia do express some known or potential components of regulated vesicular exocytosis, including synapsin I, synaptotagmins XI and XVI, syntaxins, snapin [74], rim2 [75], and Lgi3 [76] (a recently identified syntaxin interactor and potential regulator of exocytosis) suggests that, unlike cortical astrocytes, Bergmann glia *in vivo* may possess the machinery for regulated release of glutamate and/or possibly other neurotransmitters.

While much of the recent focus on glia has been on their novel roles, it is worth noting that our knowledge of the molecular mechanisms remains incomplete even for the oldest and most commonly-associated role of glia: serving as “nerve glue,” a term coined by Rudolf Virchow in 1859. In this context, it is interesting that one of the largest set of genes we found expressed at high levels in Bergmann glia consists of cell adhesion molecules and receptors known or hypothesized to mediate cell-cell or cell-ECM binding. These include well-established adhesion molecules and receptors such as brevican, tenascin C, integrin αv, and dystroglycan-1, some of which are critical for structural integrity of the glial scaffold [77]. A less studied adhesion molecule that we find in Bergmann glia, *Chl1*, was also shown recently to be important for the guidance and stabilization of stellate cell arbors projecting onto Purkinje cell dendrites [78], highlighting the important role of glial cell adhesion molecules in the development and maintenance of neuronal connections. In light of these findings, we believe that the putative glial adhesion molecules we identify, including cadherin 22, CD164 and junction adhesion molecule 2 (*Jam2*), merit further investigation.

In addition to identifying possible molecular players in known functions of Bergmann glia, the genes emerging from our study also strengthen the possibility of novel roles of these cells. Recently, two studies identified putative neural stem cells in the postnatal cerebellum [45], [46]. While the identities of these cells remain unknown, a hypothesis has emerged that perhaps Bergmann glia could be these stem cells [44]. A study from the same lab found that two transcription factors that regulate neural stem cell identity, *Sox1* and *Sox2*, are found in postnatal Bergmann glia [79]. We now significantly expand this line of inquiry by examining the full expression profiles of Bergmann glia and identifying additional genes that have previously been implicated in “stem-ness” of neural stem cells [24]. The molecular and morphological changes that Bergmann glia undergo in response to injury, granule cell death, or implantation of embryonic granule cell precursors indicate that they remain highly plastic [80]. Whether they possess the latent genetic potential to serve as neural precursors and could do so in response to an appropriate stimulus remains a tantalizing possibility. Furthermore, the mechanisms that may normally repress this potential in the adult cerebellum merit further investigation. In this regard, our observation that the Bone Morphogenic Protein antagonist, *Nbl1* (neuroblastoma, suppression of tumorigenicity 1) shows highly elevated expression in adult Bergmann glia compared to P6 is interesting, since this gene has been shown previously to repress maintenance of the precursor state and promote neuronal differentiation through its action on BMP7 [81].

## Materials and Methods

### Ethics Statement

Experiments were performed in accordance with National Institutes of Health guidelines for the care and use of laboratory animals, and with approval of the Animal Care and Use Committee of Children's Hospital Boston.

### Isolation of Single Bergmann Glial Cells

Mice of ages P6 and P30 expressing GFP under the control of the GFAP promoter (GFAP-GFP mice) were used. From GFAP-GFP mouse brains, slices of the mid-sagittal third of the cerebellum were cut in cold Hank's Balanced Salt Solution (HBSS). By cutting only from the mid-sagittal region, where Bergmann glial processes run mostly parallel to the sagittal plane, damage to glial processes was minimized. Furthermore, isolation of Bergmann glia from a restricted region of the cerebellum should minimize developmental heterogeneity between individual cells. Slices were cut into smaller pieces in cold Ca^++^- and Mg^++^-free HBSS containing 10 mM HEPES; The tissues were incubated in papain (20 U/ml), and DNase I (20 U/ml) in Ca^++^- and Mg^++^-free HBSS on a shaker for ∼30 min at 37°C. The protease solution was then replaced with Hanks Balanced Salt Solution (HBSS) containing 1 mg/ml albumin ovomucoid protease inhibitor, and the tissue was gently triturated using fire-polished glass pipettes of decreasing bore diameter. Cells were pelleted by centrifugation and resuspended in cold HBSS. A small aliquot of the cell suspension was added to a Petri dish with cold Ca^++^- and Mg^++^-free HBSS and individual cells were harvested by mouth pipetting into pulled glass microcapillaries attached to a micromanipulator. Bergmann glia were recognized by their GFP fluorescence and morphology. Each picked cell was rinsed in a fresh dish with HBSS and re-picked with a new microcapillary. Harvested cells were immediately seeded into PCR tubes containing reverse transcription buffer, and placed on ice. In control experiments, single GFP-negative cells and putative astrocytes were also picked. Astrocytes from cerebella of GFAP-GFP mice showed weaker GFP staining compared to Bergmann glia and lacked the characteristic unipolar processes of the latter. The identity of the different cerebellar cells was always verified subsequently by PCR and Southern blot.

### Single-Cell RT-PCR and Microarray Hybridization

Single-cell RT-PCR was performed as described previously [25], [27], [82]. Briefly, amplified cDNA was synthesized by lysing the cell, reverse transcribing the cell RNA after oligo-dT priming, poly-A tailing the 5′end of the cDNA, and finally amplifying the cell cDNA with a unique poly-T primer (AL1: ATTGGATCCAGGCCGCTCTGGACAAAATATGAATTC(T)24). The reverse transcription was performed in limiting conditions of nucleotides and time in order to generate cDNAs of uniform size (∼0.5 to 1 kb), which are more likely to be uniformly amplified and to accurately reflect the relative abundances of various mRNAs in the cell. After 50 cycles of PCR, several micrograms of cDNA were generated from each cell. Five µl of the cDNA was run on a 1.5% agarose gel to verify the presence of a smear from ∼0.5 to 1 kb. Using this original cDNA as template, additional cDNA could be faithfully reamplified as necessary by PCR using the AL1 primer, as described previously [82]. Southern blots for several ubiquitous and cell-specific marker genes were then performed as described [82], [83] to assess the quality and representation of the single cell cDNA, and to verify cell identity. *Actb* and *Actg* were used as high and medium abundance markers, respectively; and *Odc* and *Ppp1ca* as low abundance markers [84]. *Gfap*, *Fabp7*, *Sept4* and *Slc1a3* were used as glial markers. Finally, the presence of *Nefl*, *Mtap2*, and in some cases also tubulin β-III was checked to detect any contaminating neurons. Only the best single cell cDNAs (10 µg of each), as determined by RT-PCR and Southern blot, were selected for labeling and microarray hybridization. In total, over 150 putative Bergmann glia were harvested, of which ∼90 yielded good cDNA smears after RT-PCR. The activity of the reverse transcriptases appeared to be the most variable factor, with noticeable lot-to-lot differences. Seventy-four of the single cell cDNAs showed robust expression of *Actb*, and of these, 31 were positive for *Actg*, *Odc* and *Ppp1ca* as well. Sixteen cDNA samples passed all quality control criteria, including presence of all tested astroglial markers and absence of all tested neuronal markers. Of these, five P6 and five P30 samples that exhibited the best quality control parameters on Affymetrix Test3 arrays were then hybridized to Affymetrix GeneChip Mouse Genome 430 2.0 microarrays at the Harvard Biopolymers Facility using standard Affymetrix protocols.

### Data Analysis of Affymetrix Gene Chips

Analyses of individual microarrays and comparisons between P6 and adult were performed using GeneSpring GX 7.3 (Agilent). Raw CEL files were processed using the RMA (Robust Multichip Average) normalization algorithm as implemented in GeneSpring GX 7.3. Normalization was performed using default settings, which included data transformation (RAW values of less than 0.01 were set to 0.01), per chip normalization to the median (each measurement was divided by the 50th percentile of all measurements in that sample), and per gene normalization (the raw expression level of each gene was divided by the median of its measurements in all samples). For statistical analysis, one-way ANOVA was performed with multiple testing correction using Benjamini and Hochberg false discovery rate (FDR) set at 0.05. To identify genes expressed at higher levels at P6 compared to P30, two separate analyses were performed. First, we selected for genes that showed over three-fold higher normalized expression at P6 compared to P30 (and additionally, met statistical criteria mentioned above and were present at RAW levels of >100 in at least four out of five P6 samples). Second, we selected for genes that were flagged “present” in P6 samples (at least four out of five) and “absent” in P30 samples (at least four out of five) (and, like above, were statistically significant and present at raw signal values >100 in P6 samples). Similar analyses were performed to identify genes that were expressed at higher levels at P30 than at P6. Finally, by using an expression level filter, highly expressed genes showing raw signal values of over 2000 in at least 4 out of 5 samples of each age (total 8 of 10) were also identified. Data for annotation and functional classification of genes was obtained through Genespring (Agilent), the Gene Ontology Consortium [85], Aceview (www.aceview.org) [86], and previous studies. Our microarray data is MIAME compliant and all raw data files have been deposited in the Gene Expression Omnibus (GEO) repository, a MIAME compliant database.

### *In Situ* Hybridization

*In situ* hybridization was performed essentially as described previously [87]. Briefly, DNA templates for transcribing cRNA probes were generated by PCR. The primers contained SP6 (in forward primers) and T7 (in reverse primers) RNA polymerase binding sequences. For *Gpr126*, the following two primer pairs were used, both of which yielded similar results:

1. Forward, 5′-ATTTAGGTGACACTATAGAAGTGAGTGGTGGAGTCCTATTCATGG-3′; reverse, 5′- TAATACGACTCACTATAGGGAGACTCTGCTGAGGTGAATCTTAGTC-3′.

2. Forward, 5′- ATTTAGGTGACACTATAGAAGTGATGGATCAGACTGTGGCATACAAG-3′; reverse, 5′- TAATACGACTCACTATAGGGAGAGTCCAGGTTGCTAAAGAATGAATG-3′. Underlined regions correspond to the SP6 (in forward primers) and T7 (in reverse primers) RNA polymerase binding sequences. ^33^P-labeled sense and antisense riboprobes were generated using SP6 and T7 polymerases respectively (Promega) and a reaction mix containing ^33^P-UTP (Perkin Elmer). Radioactive *in situ* hybridization was performed on 16 µm brain cryosections as described previously [88], [89].

### Gene Expression Atlases

Several online reference atlases of mRNA expression were used to corroborate the expression of genes identified in the microarrays. These included the Allen Brain Atlas (ABA) (http://www.brain-map.org/) [90], the Brain Gene Expression Map (http://www.stjudebgem.org) [91], and GenePaint (http://www.genepaint.org) [92].

## Supporting Information

Table S1 Developmentally-regulated Bergmann glia genes (P6>P30 by >3-fold, or present at P6 and absent at P30)
(0.11 MB XLS)

Table S2 Developmentally-regulated Bergmann glia genes (P30>P6 by >3-fold, or present at P30 and absent at P6)
(0.04 MB XLS)

Table S3 Presumptive neuron-specific genes seen in Bergmann glial cDNA samples
(0.02 MB XLS)

Table S4 Genes expressed at moderate to high levels in Bergmann glia (P6 and P30) (mean raw signal values >2000)
(1.24 MB XLS)

Table S5 Complete data set of 45,101 probe sets for all Gene Chips used in our single cell gene expression analysis
(9.52 MB ZIP)

Figure S1 Myelin-related genes are expressed in adult Bergmann glia. Mid-sagittal views of adult mouse cerebella with *in situ* hybridization images (left panels) and expression level analysis (right panels), as obtained from the Allen Brain Atlas. Insets in all images are from the dorso-rostral region of lobule V (asterisk in top panels). Top row, mRNA for *Pmp22*, a constituent of myelin, is expressed most strongly in putative oligodendrocytes in the white matter (arrows). Surprisingly, however, there is also signal in the Purkinje cell layer (PCL), in a pattern consistent with expression in Bergmann glia (arrowheads). Second row, *Plp1*, the major constituent of CNS myelin, exhibits a similar expression pattern. Although the strongest staining is in putative oligodendrocytes (arrows), there is also distinct signal in Bergmann glia (arrowheads). Third row, *Lgi4*, which has been shown to be important for myelination in the PNS, shows robust and Bergmann glia-specific expression in the cerebellum. Fourth row, *Mpzl1*, another gene thought to be involved in myelination, is expressed in at least a subset of Bergmann glia (arrowheads). Scale bar, 500 µm in all panels, 140 µm in insets.
(4.55 MB TIF)

Figure S2 Bergmann glia-specific gene expression in the adult cerebellum. Mid-sagittal views of adult mouse cerebella with *in situ* hybridization images (left panels) and expression level analysis (right panels), as obtained from the Allen Brain Atlas. Insets in all images are from the dorso-rostral region of lobule V as in Fig. S1. *Luzp2*, *Gpr89*, *Lgi4* and *Gdf10* all appear to be expressed very specifically in the Purkinje cell layer in the adult cerebellum. This restricted localization, along with a cellular expression pattern in the PCL that matches those of well established astroglial markers (Fig. S3), suggests that these genes are Bergmann-glia specific in the adult cerebellum and may serve as novel markers for these cells. Scale bar, 500 µm in all panels, 140 µm in insets.
(4.22 MB TIF)

Figure S3 Expression patterns of well established astroglial markers in the adult cerebellum. Mid-sagittal views of adult mouse cerebella with *in situ* hybridization images (left panels) and expression level analysis (right panels), as obtained from the Allen Brain Atlas. Insets in all images are from the dorso-rostral region of lobule V as in Fig. S1. The glial genes, *Slc1a3*, *Fabp7*, *S100β* and *Sept4* are four widely used astroglial markers, and their expression in Bergmann glia (but not Purkinje cells or other cerebellar neurons) has been confirmed by previous studies. The cellular expression patterns of these genes are presented here to serve as controls against which the Bergmann glial expression of new genes (for example, as in Fig. S2) can be compared. Scale bar, 500 µm in all panels, 140 µm in insets.
(4.27 MB TIF)
