# Supplementary figures and images for: Identification of Novel Glial Genes by Single-Cell Transcriptional Profiling of Bergmann Glial Cells from Mouse Cerebellum
Source: PLoS One. 2010 Feb 12;5(2):e9198. doi: 10.1371/journal.pone.0009198 (PMC2820553; doi:10.1371/journal.pone.0009198)

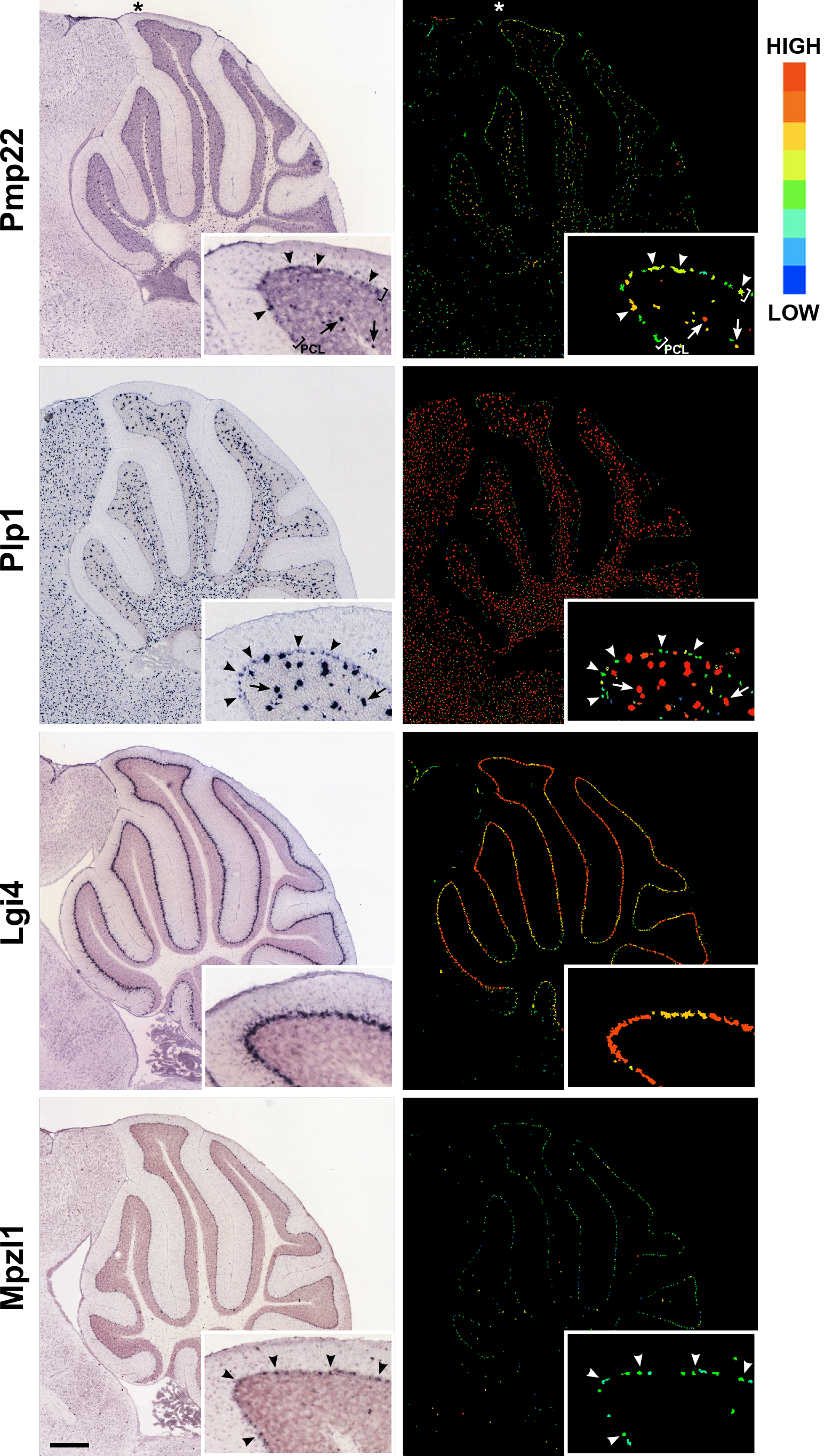

Supplement: Figure S1 — Myelin-related genes are expressed in adult Bergmann glia. Mid-sagittal views of adult mouse cerebella with in situ hybridization images (left panels) and expression level analysis (right panels), as obtained from the Allen Brain Atlas. Insets in all images are from the dorso-rostral region of lobule V (asterisk in top panels). Top row, mRNA for Pmp22, a constituent of myelin, is expressed most strongly in putative oligodendrocytes in the white matter (arrows). Surprisingly, however, there is also signal in the Purkinje cell layer (PCL), in a pattern consistent with expression in Bergmann glia (arrowheads). Second row, Plp1, the major constituent of CNS myelin, exhibits a similar expression pattern. Although the strongest staining is in putative oligodendrocytes (arrows), there is also distinct signal in Bergmann glia (arrowheads). Third row, Lgi4, which has been shown to be important for myelination in the PNS, shows robust and Bergmann glia-specific expression in the cerebellum. Fourth row, Mpzl1, another gene thought to be involved in myelination, is expressed in at least a subset of Bergmann glia (arrowheads). Scale bar, 500 µm in all panels, 140 µm in insets. (4.55 MB TIF) [file pone.0009198.s006.tif]

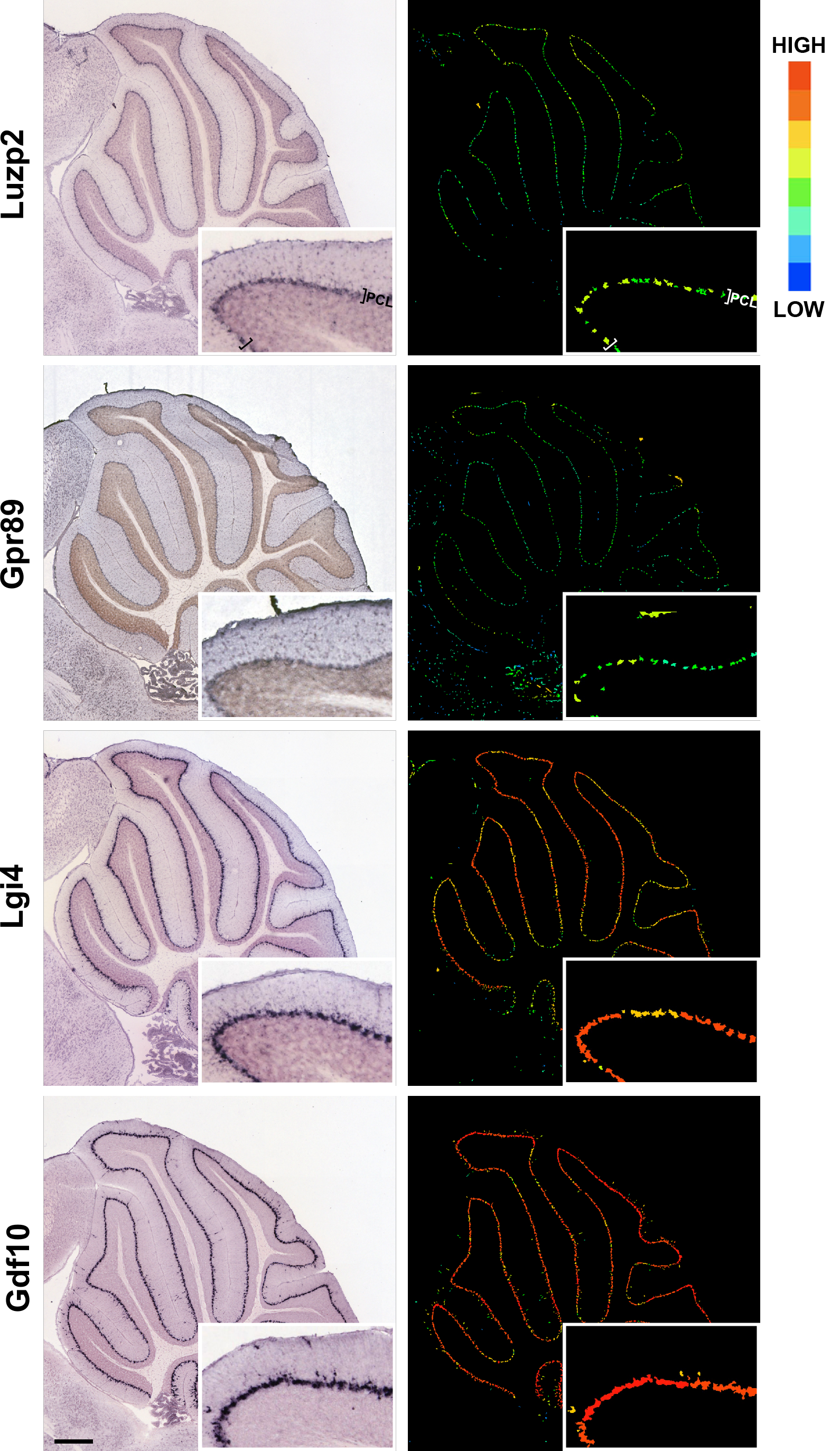

Supplement: Figure S2 — Bergmann glia-specific gene expression in the adult cerebellum. Mid-sagittal views of adult mouse cerebella with in situ hybridization images (left panels) and expression level analysis (right panels), as obtained from the Allen Brain Atlas. Insets in all images are from the dorso-rostral region of lobule V as in Fig. S1. Luzp2, Gpr89, Lgi4 and Gdf10 all appear to be expressed very specifically in the Purkinje cell layer in the adult cerebellum. This restricted localization, along with a cellular expression pattern in the PCL that matches those of well established astroglial markers (Fig. S3), suggests that these genes are Bergmann-glia specific in the adult cerebellum and may serve as novel markers for these cells. Scale bar, 500 µm in all panels, 140 µm in insets. (4.22 MB TIF) [file pone.0009198.s007.tif]

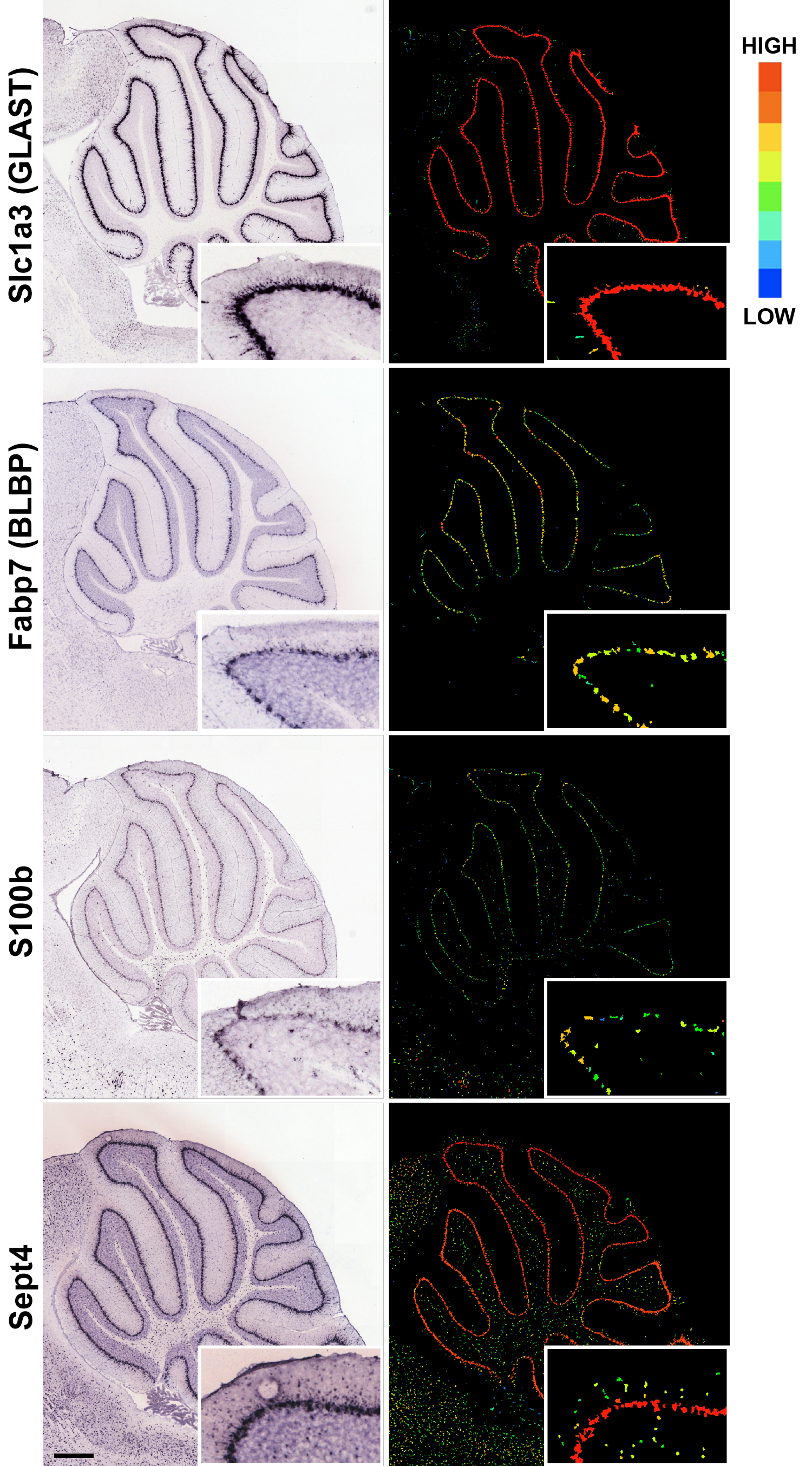

Supplement: Figure S3 — Expression patterns of well established astroglial markers in the adult cerebellum. Mid-sagittal views of adult mouse cerebella with in situ hybridization images (left panels) and expression level analysis (right panels), as obtained from the Allen Brain Atlas. Insets in all images are from the dorso-rostral region of lobule V as in Fig. S1. The glial genes, Slc1a3, Fabp7, S100β and Sept4 are four widely used astroglial markers, and their expression in Bergmann glia (but not Purkinje cells or other cerebellar neurons) has been confirmed by previous studies. The cellular expression patterns of these genes are presented here to serve as controls against which the Bergmann glial expression of new genes (for example, as in Fig. S2) can be compared. Scale bar, 500 µm in all panels, 140 µm in insets. (4.27 MB TIF) [file pone.0009198.s008.tif]
